# The association between gout and subsequent cardiovascular events: a retrospective cohort study with 132,000 using propensity score matching in primary care outpatients in Germany

**DOI:** 10.1007/s00392-024-02537-9

**Published:** 2024-09-10

**Authors:** Jamschid Sedighi, Mark Luedde, Julia Gaensbacher-Kunzendorf, Samuel Sossalla, Karel Kostev

**Affiliations:** 1 Medical Clinic I, Cardiology and Angiology, Justus-Liebig-University, Klinikstraße 33, 35392 Giessen, Germany; 2 Department of Cardiology, Angiology and Intensive Care Medicine, University Medical Center of Schleswig Holstein, Campus Kiel, Kiel, Germany; 3 Christian-Albrechts-University of Kiel, Kiel, Germany; 4 Cardiologicum Bremerhaven, Bremen, Germany; 5 Department of Cardiology, Kerckhoff-Clinic, Bad Nauheim, Germany; 6 German Center for Cardiovascular Research (DZHK), Partner Site Rhine-Main, Frankfurt, Germany; 7 Epidemiology, IQVIA, Main Airport Center, Unterschweinstiege 2–14, 60549 Frankfurt am Main, Germany

**Keywords:** Goat, Sex-related differences, Cardiovascular events, Prevention

## Abstract

**Background:**

Both the risk of developing heart disease and the course of the disease are determined in particular by comorbidities. In this context, gout has recently been identified as an important factor in influencing the development of cardiovascular events such as heart failure or coronary artery disease.

**Methods:**

This retrospective cohort study compared the incidence of angina pectoris (AP) (ICD-10: I20), myocardial infarction (MI) (ICD-10: I21, I22), chronic coronary heart disease (CHD) (ICD-10: I25), atrial fibrillation (AF), and heart failure (HF) as a function of gout in Germany in a large collective of 66,000 gout patients in comparison to 66,000 individuals without gout between using propensity score matching (1:1) from January 2005 to December 2020.

**Results:**

Within 10 years after the index date, AP was diagnosed in 5.2% of gout and 2.9% of non-gout patients (*p* < 0.001), MI in 3.1% of gout and 2.2% of non-gout patients (*p* < 0.001), CHD in 16.5% of gout and 11.8% of non-gout patients, AF in 12.6% of gout and 8.4% of non-gout patients (*p* < 0.001), and HF in 14.7% of gout and 8.5% of non-gout patients (*p* < 0.001). For all diagnoses except CHD, the association was stronger in male than in female patients.

**Conclusion:**

The relationship shown between gout and cardiovascular disease indicates that gout could be one of a series of inflammatory conditions that increase the risk of cardiac disease. The association we have shown between gout and all major cardiac diseases suggests that there is a risk modifier, the treatment of which could help prevent these diseases. Further research is needed to determine whether treating gout can effectively reduce this risk.

**Supplementary Information:**

The online version contains supplementary material available at 10.1007/s00392-024-02537-9.

## Introduction

Both the risk of developing heart disease and the course of the disease are determined in particular by comorbidities [1, 2]. In this context, gout has recently been identified as an important factor in influencing the development of cardiovascular events such as heart failure [3] or coronary artery disease [4, 5].

In the present study, we investigated the incidence of a broad spectrum of cardiovascular diseases (angina pectoris (AP), myocardial infarction (MI), chronic coronary heart disease (CHD), atrial fibrillation (AF) and heart failure (HF)) in a large collective of 66,000 gout patients in comparison to 66,000 individuals without gout.

## Methods

This retrospective cohort study is based on data from the Disease Analyzer database (IQVIA), which contains drug prescriptions, diagnoses, and basic medical and demographic data obtained directly and in anonymous format from computer systems used in the practices of general practitioners and specialists [6]. Covering approximately 3% of all private practices in Germany, the database can justifiably be described as representative of general and specialized practices in Germany [6]. This study included adult patients (≥ 18 years) with an initial diagnosis of gout (ICD-10: M10) in 1284 general practices in Germany between January 2005 and December 2020 (index date; Supplementary Fig. 1). Further inclusion criteria included an observation time of at least 12 months prior to the index date and a follow-up time of at least 6 months after the index date. Patients with diagnoses of ischemic heart disease (ICD-10: I20–I25), I48.0, I48.1, I48.2, I48.9) or heart failure (ICD-10: I50) prior to or on the index date were excluded.

Differences in the sample characteristics and diagnosis prevalence between gout and non-gout cohorts were compared using the Wilcoxon signed-rank test for continuous variables, the McNemar test for categorical variables with two categories, and the Stuart–Maxwell test for categorical variables with more than two categories.

The 10-year cumulative incidence of AP; MI; CHD; AF and HI in the cohort with and without gout was further studied with Kaplan–Meier curves, and these curves were compared using the log-rank test. P value of < 0.01 was considered statistically significant. Analyses were carried out using SAS version 9.4 (SAS Institute, Cary, USA).

**Fig. 1:**
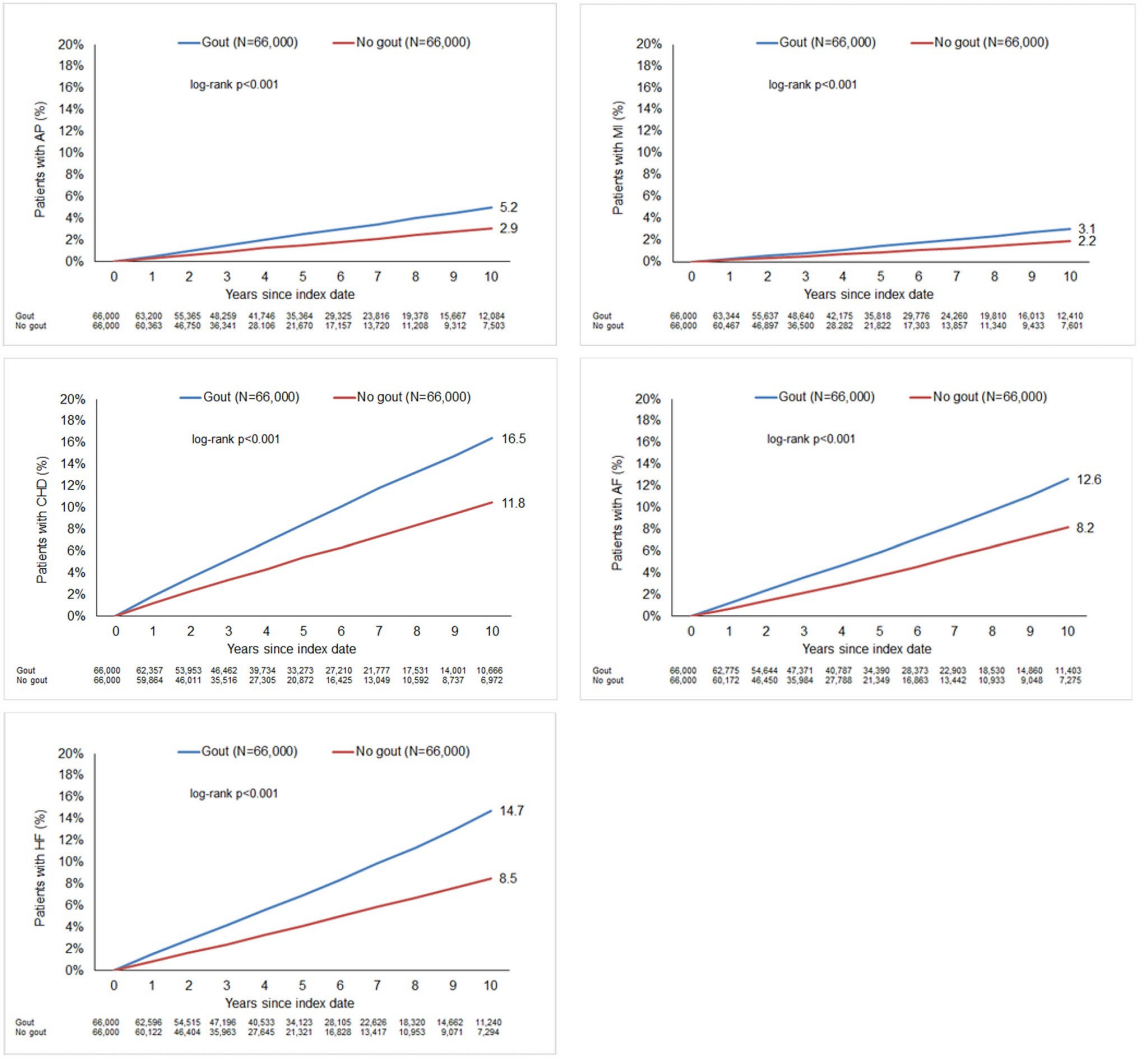
10-Year-cumulative incidence of angina pectoris, myocardial infarction, coronary heart disease, atrial fibrillation, and heart failure in patients with and without gout

After applying similar inclusion criteria, individuals without gout were matched to gout patients using propensity score matching (1:1) based on sex, age, yearly consultation frequency during the follow-up, diabetes, obesity, lipid metabolism disorders, hypertension, chronic bronchitis/chronic obstructive pulmonary disease (COPD), cancer, rheumatoid arthritis and osteoarthritis diagnoses prior to the index date. For the non-gout cohort, the index date was that of a randomly selected visit between January 2005 and December 2020 (Fig. 1). Diabetes (ICD-10: E10–E14), obesity (ICD-10: E66), lipid metabolism disorders (ICD-10: E78), and hypertension (ICD-10: I10) were included as these diagnoses are strongly associated with heart diseases.

## Results

The basic characteristics of study patients are displayed in Table 1. The mean age was 59 years; 30% were women. Patients visited their GPs an average of 6.6 times per year during the follow-up period. Within 12 months prior to the index date, a much higher proportion of gout patients received diuretic prescriptions than those without gout (28.5% vs. 19.3%). Of the 66,000 gout patients, 71% had a diagnosis of unspecified gout (ICD-10: M10.9) and 27% idiopathic gout (ICD-10: M10.0). Other gout types were extremely rare.

**Table 1:** Baseline characteristics of the study sample (after propensity score matching)

Variable	Proportion among gout patients (%) *N* = 66,000	Proportion among non-gout patients (%) *N* = 66,000	*p* value
Age (mean, SD)	59.3 (14.4)	59.4 (14.5)	0.305
Age 18–50	26.3	26.2	0.484
Age 51–60	25.9	25.8	
Age 61–70	23.4	23.4	
Age 71–80	18.3	18.3	
Age > 80	6.1	6.3	
Women	30.3	30.3	1.000
Men	69.7	69.7	
Number of physician visits per year during the follow-up (mean, SD)	6.6 (4.6)	6.6 (4.6)	0.991
Diabetes	21.6	21.4	0.228
Obesity	15.3	15.1	0.167
Lipid metabolism disorders	37.2	36.9	0.284
Hypertension	58.9	58.9	0.996
Chronic bronchitis/COPD	10.7	10.7	0.993
Cancer	8.4	8.6	0.192
Rheumatoid arthritis	2.7	2.3	< 0.001
Osteoarthritis	23.9	18.8	< 0.001
Drug classes prescribed within 12 months prior to the index date			
Diuretics	28.5	19.3	< 0.001
Beta blockers	25.3	20.1	< 0.001
Calcium channel blockers	13.5	12.6	< 0.001
ACE inhibitors	23.8	22.7	< 0.001
Angiotensin II receptor blockers	19.0	16.1	< 0.001
Statins	14.6	15.9	< 0.001
Aspirin	5.4	5.5	0.258

Proportions of patients in % given, unless otherwise indicated

*SD* standard deviation

Within 10 years after the index date, AP was diagnosed in 5.2% of gout and 2.9% of non-gout patients (*p* < 0.001), MI in 3.1% of gout and 2.2% of non-gout patients (*p* < 0.001), CHD in 16.5% of gout and 11.8% of non-gout patients, AF in 12.6% of gout and 8.4% of non-gout patients (*p* < 0.001), and HF in 14.7% of gout and 8.5% of non-gout patients (*p* < 0.001) (Fig. 1).

In the regression analysis, we found a strong association between gout and subsequent cardiovascular diseases. The strongest association was observed for AP (HR 1.61; 95% CI 1.49–1.74) and HF (HR 1.61; 95% CI 1.53–1.69), followed by AF (HR 1.40; 95% CI 1.33–1.47), CHD (HR 1.37; 95% CI 1.32–1.43), and MI (HR 1.36; 95% CI 1.23–1.50). For all diagnoses except CHD, the association was stronger in male than in female patients (Table 2).

**Table 2:** Association between gout and subsequent cardiovascular diseases in patients followed in general practices in Germany (multivariable Cox regression models)

Outcome diagnosis	Adjusted HR for gout (95% CI)	*p* value
Full cohorts *n* = 66,000		
Angina pectoris	1.61 (1.49–1.74)	< 0.001
Myocardial infarction	1.36 (1.23–1.50)	< 0.001
Coronary heart disease	1.37 (1.32–1.43)	< 0.001
Atrial fibrillation	1.40 (1.33–1.47)	< 0.001
Heart failure	1.61 (1.53–1.69)	< 0.001
Women *n* = 19.998		
Angina pectoris	1.53 (1.33–1.77)	< 0.001
Myocardial infarction	1.29 (1.05–1.59)	0.017
Coronary heart disease	1.42 (1.31–1.54)	< 0.001
Atrial fibrillation	1.29 (1.18–1.41)	< 0.001
Heart failure	1.55 (1.44–1.68)	< 0.001
Men *n* = 46,002		
Angina pectoris	1.64 (1.49–1.80)	< 0.001
Myocardial infarction	1.37 (1.25–1.53)	< 0.001
Coronary heart disease	1.36 (1.29–1.42)	< 0.001
Atrial fibrillation	1.46 (1.37–1.55)	< 0.001
Heart failure	1.66 (1.56–1.76)	< 0.001

## Discussion

The relationship shown between gout and cardiovascular disease indicates that gout could be one of a series of inflammatory conditions that increase the risk of cardiac disease (for example, rheumatoid arthritis). The prevalence of rheumatoid arthritis was higher in the gout group (2.7%) than in the non-gout population (2.3%), as observed in our study.

On the other hand, it is also possible that this link we observed was due to specific effects of elevated uric acid levels [7]. The molecular signaling pathways involved need to be further deciphered. The association we have shown between gout and all major cardiac diseases suggests that there is a risk modifier, the treatment of which could help prevent these diseases [8]. However, no clear protective effect of, e.g., allopurinol for the prevention of cardiovascular endpoints has yet been demonstrated in gout patients [9]. Although initial evidence suggested that cardiovascular patients might benefit from allopurinol even in the absence of gout, an important new study has shown that patients with ischemic heart disease, for example, do not benefit from taking allopurinol [10].

Our study lends support to the findings of McDowell et al. that elevated uric acid (UA) levels, which are associated with gout, increase the risk of cardiovascular disease. Elevated uric acid levels contribute to oxidative stress and vascular dysfunction, thereby establishing a link between gout and adverse cardiovascular outcomes [11]. Although reducing uric acid (UA) levels via sodium-glucose co-transporter 2 (SGLT2) inhibitors such as dapagliflozin has demonstrated benefits, UA appears to be more of a marker of disease severity than a direct risk factor [12]. Further research should be conducted to gain a more detailed understanding of the comorbidities associated with gout and to investigate alternative treatments that target oxidative stress, with the aim of improving the management of cardiovascular risk in patients with gout.

Better knowledge of the molecular and genetic links between gout and cardiovascular disease may also help explain the sex-related differences we found. These appear to be disease-specific: While there is a stronger association between gout and disease risk in women, as consistent with previous studies in CHD [13], the risk for the other cardiac diseases is higher in men.

The observed sex-related differences in the association between gout and cardiovascular events may be attributed to a number of biological, hormonal, and behavioral factors. Hormonal differences, such as the protective effects of estrogen against cardiovascular disease in premenopausal women, may be a significant contributing factor. Following menopause, a decline in estrogen levels may elevate the risk of cardiovascular disease in women, potentially modifying the influence of gout [13, 14]. It is postulated that males with elevated testosterone levels may exhibit augmented uric acid production, thereby contributing to a higher prevalence of gout and an elevated risk of cardiovascular disease. Additionally, biological differences in uric acid metabolism and higher baseline uric acid levels in men may contribute to the stronger observed association between gout and cardiovascular events in men [14, 15].

Behavioral and lifestyle factors, such as diet, alcohol consumption, and physical activity, differ between men and women and may modify the impact of gout on cardiovascular health.

It is crucial to distinguish between risk factors and risk modifiers in the context of our study. In the context of disease development, risk factors are defined as variables that directly increase the likelihood of a particular disease occurring. For example, hypertension, smoking, and high cholesterol are well-established risk factors for cardiovascular disease, as they directly contribute to the pathophysiology of the condition.

Conversely, risk modifiers impact the influence of these risk factors on disease outcomes. In our study, gout serves as a risk modifier. While gout itself may not directly cause cardiovascular events, its presence can exacerbate the effects of existing cardiovascular risk factors, leading to an increased likelihood of adverse cardiovascular outcomes. This distinction is crucial for understanding the role of gout in the context of cardiovascular health and for developing targeted management strategies for patients with gout.

Our study relates more to the overarching aspect of the relationship between gout and cardiovascular disease in general. Understanding the association between gout and cardiovascular risk has significant implications for clinical practice. If gout is identified as a risk modifier for cardiovascular events, healthcare providers can better monitor and manage cardiovascular risk factors in patients with gout. This could lead to improved patient outcomes through more targeted and effective management strategies.

## Limitation of the study

Our study is subject to a number of limitations which have to be borne in mind: Our data refer to ICD-10 codes only, there are no uric acid level measurements in our database that would allow for the identification of, for example, a correlation between uric acid levels and cardiovascular events. Furthermore, the use of the ICD-10 coding system may lead to misclassification and undercoding of certain diagnoses. It is important to note, however, that the ICD codes used do not reflect the severity of these comorbidities. For example, the term "obesity" encompasses both individuals with a body mass index (BMI) of 27 kg/m^2^ who are overweight and those with a BMI of 42 kg/m^2^ who are severely obese. Furthermore, discrepancies in concomitant medications, which were not fully addressed, could influence the study outcomes. These limitations should be addressed to facilitate a more comprehensive interpretation of the findings. Future studies should aim to incorporate more detailed data on comorbidity severity and medication use to enhance understanding of the impact of these factors on the association between gout and cardiovascular risk.

Despite the use of propensity score matching to adjust for cardiovascular risk modifier, patients with gout received a greater number of cardiovascular drugs than those without gout. This suggests a higher prevalence of existing cardiovascular disease or more severe risk factors that are not fully captured by ICD codes. Conversely, patients without gout were more often treated with statins, suggesting a lower baseline cardiovascular risk. These differences in medication use underscore the necessity of considering treatment regimens when interpreting the association between gout and cardiovascular risk. Furthermore, we have no mortality data.

Another important limitation concerns the study design, which is based on retrospective database analyses from patient in Germany. However, thanks to the size of the collective examined and the extensive data set from the Disease Analyzer Database (IQVIA), our study provides a good overview of general practitioner in Germany. Additionally, the use of propensity score matching to adjust for multiple cardiovascular risk factors and the comprehensive medical and demographic information available contribute to the study's robustness. These strengths may stimulate further studies on the association between gout and cardiovascular events.

## Supplementary Information

Below is the link to the electronic supplementary material.

Supplementary file1 (PDF 173 kb)
