# Supplementary figures and images for: The association between gout and subsequent cardiovascular events: a retrospective cohort study with 132,000 using propensity score matching in primary care outpatients in Germany
Source: Clin Res Cardiol. 2024 Sep 10;114(9):1185–90. doi: 10.1007/s00392-024-02537-9 (PMC12408739; doi:10.1007/s00392-024-02537-9)

**Supplementary Figure 1** Selection of study patients

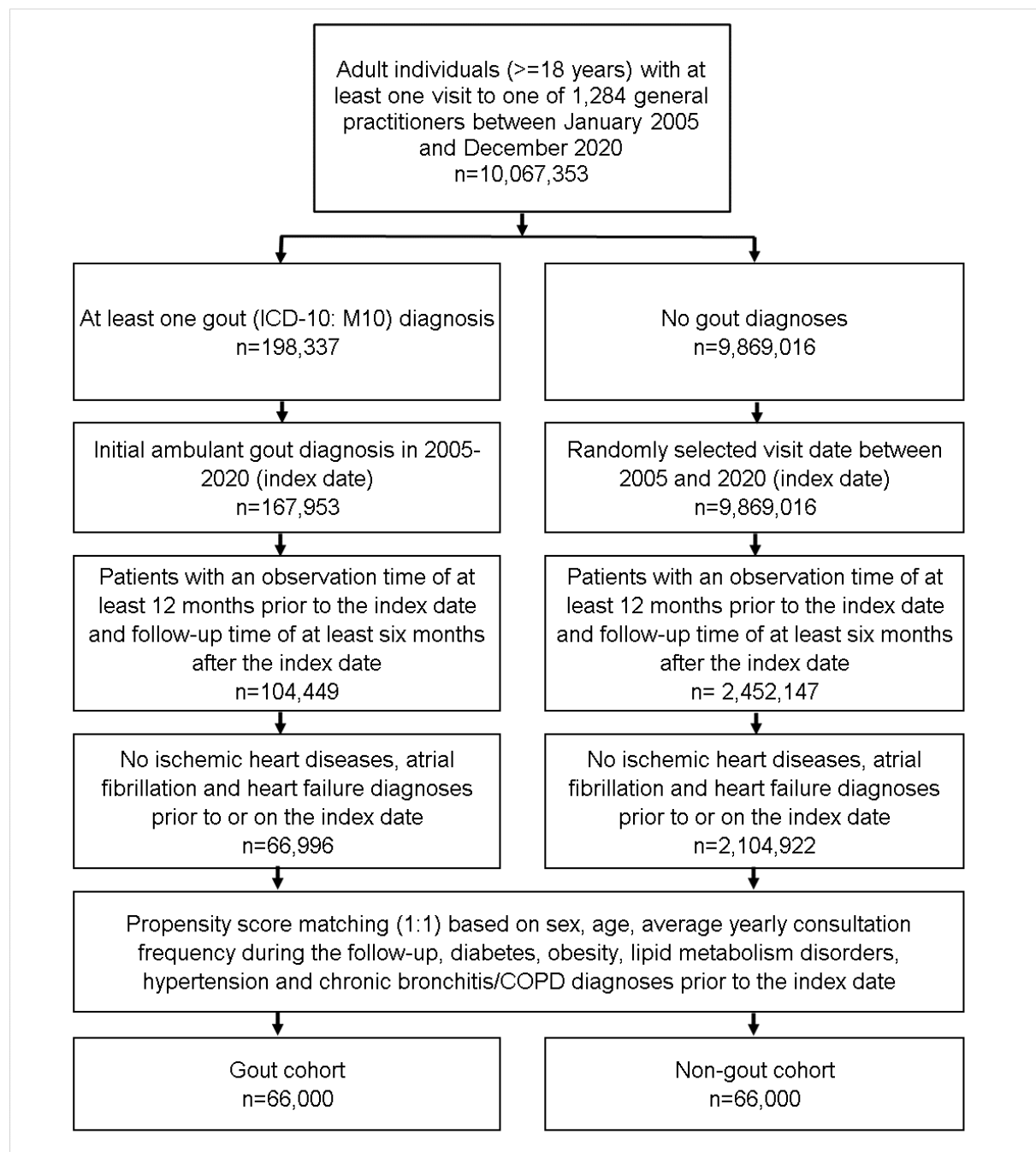

Supplement: Supplementary file 1 — Supplementary file1 (PDF 173 kb) [file 392_2024_2537_MOESM1_ESM.pdf]
